# Vaccination against *Streptococcus pneumoniae* in Children Born between 2015 and 2018 in Poland—How Has the Introduction of Free Compulsory Pneumococcal Vaccination Affected Its Uptake?

**DOI:** 10.3390/vaccines11111654

**Published:** 2023-10-28

**Authors:** Wojciech Malchrzak, Mateusz Babicki, Agnieszka Mastalerz-Migas

**Affiliations:** Department of Family Medicine, Faculty of Medicine, Wroclaw Medical University, 50-367 Wrocław, Poland; ma.babicki@gmail.com (M.B.); agnieszka.migas@gmail.com (A.M.-M.)

**Keywords:** invasive pneumococcal disease, pneumococcal vaccine, *Streptococcus pneumoniae*, vaccination program

## Abstract

Starting from 2017, pneumococcal vaccination was added to the Polish vaccination calendar as mandatory for all children born after 2016. The 10-valent conjugate vaccine was selected as mandatory and therefore free of charge. This paper aims to examine the impact of introducing mandatory vaccination on vaccine uptake. For this purpose, an analysis was conducted for 1595 vaccination record sheets from outpatient clinics in Wrocław and surrounding villages for children born 2015–2018. After the introduction of compulsory vaccination, the percentage of children fully vaccinated against *pneumococcus* increased (60.4% vs. 84.8%, *p* < 0.001). A significant decrease in the number of children who did not receive any dose of the vaccine was observed (27.8% to 3.3%, *p* < 0.001). The introduction of compulsory vaccination did not affect the completion of the pneumococcal schedule (11.8% vs. 11.9%). Compulsory PCV10 vaccination resulted in the less frequent choice of the 13-valent vaccine (72.3% vs. 19.9%, *p* < 0.001). More children in rural outpatient clinics were vaccinated against *pneumococcus* compared to urban outpatient clinics (84.8% vs. 70.8%, *p* < 0.001). The introduction of free pneumococcal vaccination increased the proportion of children vaccinated, although it did not affect the rate of discontinuation of the initiated schedule. In Poland, the increased popularity of the 10-valent vaccine at the expense of the 13-valent one translated into a change in the proportion of pneumococcal serotypes causing invasive pneumococcal disease.

## 1. Introduction

*Streptococcus pneumoniae* is a bacterium that mainly causes non-invasive respiratory infections such as middle ear infections and pneumonia. In some cases, it can cause infection of physiologically sterile parts of the body. When this happens, the most common clinical manifestations are pneumonia with bacteremia, meningitis, or sepsis—collectively known as invasive pneumococcal disease (IPD) [1]. In 2021, 8962 cases of IPD were reported in Europe, of which 955 were in Poland [2]. In an era of ever-increasing bacterial resistance to antibiotics, their effectiveness is increasingly limited. It is, therefore, crucial to take preventive measures against IPD, and immunization still remains the most effective method of preventing the disease [1].

Currently, conjugate vaccines (PCVs) immunizing against ten (PCV10—Synflorix, GlaxoSmithKline Biologicals, Belgium) or thirteen (PCV13—Prevenar 13, Pfizer Europe MA EEIG, Belgium) *S. pneumoniae* serotypes are used for the general vaccination of children in Poland [3]. Seven-valent vaccines have been used in the past, and fifteen- and twenty-valent vaccines have also emerged in recent years, but they are not available in Poland for the pediatric population. The 20-valent vaccine has so far been registered only for adult patients. Polysaccharide (PPSV) vaccines are not suitable for the youngest children due to their inability to adequately stimulate the child’s immature immune system [4].

The Polish vaccination calendar for 2023 distinguishes between mandatory vaccinations, which are free of charge, and recommended vaccinations—for which the child’s parents must pay [3]. Until the end of 2016, pneumococcal vaccination was not mandatory in Poland. Free vaccination was available only to children from specific risk groups, including those with HIV infection, bone marrow or organ transplants, cochlear implants, or children with certain chronic diseases, as well as premature babies and children with a birth weight of less than 2500 g [5]. If parents wanted to vaccinate a child ineligible for mandatory vaccination, they had to bear the cost of the vaccination. In Poland, universal mandatory free vaccination against *S. pneumoniae* was introduced in 2017 and is continued to this day. Currently, the PCV10 vaccine is used for the general public in a 2+1 schedule (two doses of primary vaccination and one booster dose), or in some cases 3+1 (three doses of primary vaccination and one booster dose). The 3+1 schedule applies to babies born prematurely and those at risk of severe pneumococcal disease [3].

Documentation of the course of immunization is maintained by the primary healthcare facility in which the child is enrolled. Each patient has his individual vaccination record sheet, which contains information related to each vaccine administered. After the vaccination, an entry is made in the clinic’s records as well as in the child’s health record that belongs to the parents [6]. It is very difficult to assess the level of pneumococcal vaccination in Poland in the period before mandatory vaccination was introduced due to the lack of registration by state institutions. Such data was analyzed nationwide only from 2017. According to a 2022 report by the National Institute of Public Health of the National Institute of Hygiene, about 96% of children in the 2017–2018 age group have been vaccinated against *pneumococcus* [7]. According to WHO data, in 2018, the percentage of fully vaccinated children in Poland was 60%. This compares with 78% and 47% in Europe and the world, respectively [8].

This study aims to assess the effect of introducing free mandatory vaccination against *S. pneumoniae* on its uptake, as well as on decisions on the choice of formulations, and to analyze the completeness of the vaccination schedules carried out. Since the introduction of free pneumococcal vaccination in Poland, to the best of the authors’ knowledge, no similar study has been conducted before. Also, there is no nationwide data available that would provide answers to the above questions.

## 2. Materials and Methods

Data for this study was collected by analyzing the vaccination record sheets kept for children born in 2015–2018 and residing in Wrocław (a city of more than 500,000 residents) and two nearby villages. The analysis included all available vaccination record sheets for children. Inclusion criteria were that the participants had to be patients of the clinic at the time of data collection and be born between 2015 and 2018. Relevant approvals were obtained from the managers of medical facilities before the record sheets were analyzed.

The database created contained information about the date of each PCV dose and the formulation used. Information on gender and date of birth was also collected, which was necessary to determine which vaccination schedule was performed. The data contained in the database do not allow the identification of the patient. Based on the date of administration of each PCV dose, the patient’s age at the time of the dose, the interval between doses, and the total number of doses administered were determined. These data made it possible to assign patients to the following groups:

- not vaccinated;
- not fully vaccinated;
- vaccinated completely.

Patients who did not receive any dose of PCV were included in the “not vaccinated” group. The group of patients with a complete vaccination schedule consisted of children who, at the time of the vaccination study, had received the full vaccination schedule in accordance with current recommendations. These criteria differed between the periods analyzed, and their detailed description is shown in Table 1 and Table 2 [3,5,9,10]. Any child who received at least one dose of PCV but did not meet the conditions to be considered completely vaccinated was categorized as “not fully vaccinated.”

### Description of Statistics

Based on the number of doses and the interval between them, it was possible to determine whether the vaccination schedule had been carried out correctly or whether it had been interrupted. This allowed each patient to be assigned to one of three groups. The Chi-squared test was used to compare qualitative variables. Statistical significance was assumed at the level of <0.05. Calculations were performed using Statistica 13 software by TIBCO Software Inc. (Palo Alto, CA, USA).

## 3. Results

### 3.1. Characteristics of the Sample Group

**Figure 1:**
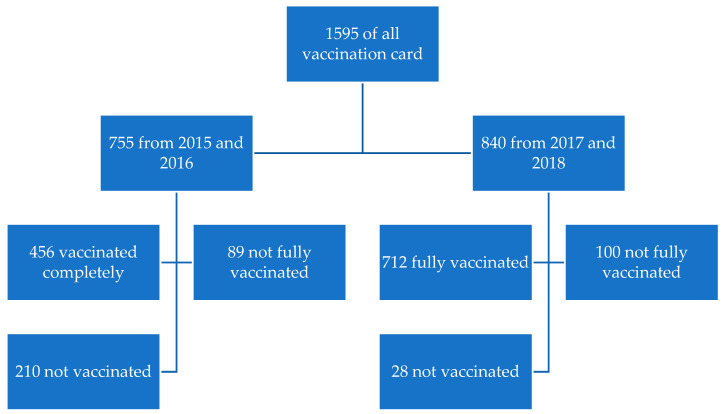
Distribution of analyzed records.

Included in the analysis were 1595 unique analyzed vaccination record sheets. Children born in 2015–2016 accounted for 47.3%, and those born in 2017–2018 accounted for 52.7%. Slightly more than half (52.2%) were women. Patients from municipal clinics accounted for 82.6% of the total. Detailed sociodemographic data is shown in Table 3. The distribution of the records is presented in Figure 1.

### 3.2. Comparison of Vaccination in the Period before and after the Introduction of Mandatory Vaccination

In the analyzed group, an increase in the percentage of children with a completed vaccination schedule was observed after the implementation of mandatory vaccination (60.4% vs. 84.8%, *p* < 0.001). What is more, the percentage of children who did not receive even one dose of PCV vaccination dropped significantly, from 27.8% to 3.3%, *p* < 0.001. The percentage of children who did not complete the vaccination schedule remained similar (11.8% vs. 11.9%).

In the analysis of preference for the formulation used, it was observed that, among children born in 2015–2016 who received at least one dose of the vaccine, the majority were vaccinated with PCV13 (72.3%), while, in 2017–2018, this percentage decreased to 19.9% in favor of PCV10, whose share was then 80.1% (*p* < 0.001). Table 4 presents information on the patients’ vaccination status, including an incomplete vaccination schedule.

In the analysis of individual sociodemographic variables on vaccination status, no significant differences were observed regarding the gender of the child (*p* = 0.989). However, a difference was observed depending on the place of residence. Patients living in rural areas were significantly more likely to be vaccinated against *pneumococcus*, with rates of 84.8% vs. 70.8% in rural and urban outpatient clinics, respectively (*p* < 0.001). There were no significant differences in the completion of the vaccination schedule depending on the vaccine used (*p* = 0.093). Table 5 shows the status of pneumococcal vaccination with uncompleted schedules according to sociodemographic data. Table 6 shows these figures broken down for 2015–2016 and 2017–2018.

An analysis was carried out in terms of the percentage of interrupted vaccinations with a particular formulation before and after the introduction of the obligation. It showed no significant differences, but, in the case of PCV10, a trend on the verge of statistical significance can be seen—after mandatory vaccination was introduced, the percentage of interrupted vaccination decreased from 16.6% to 11.7%. Details are shown in Table 7.

## 4. Discussion

The present study is based on an analysis of birth charts of children from Wrocław (Poland) and surrounding villages. For the purpose of the study, 1595 birth charts from three medical facilities of children born between 2015–2016 (755) and 2017–2018 (840) were analyzed. The number of births in the region in the period 2015–2016 was 53,432, and for 2017–2018, 55,853 newborns. This indicates that 1.5 per cent of babies born in the periods indicated were analyzed [11,12,13,14]. The results of the present study indicate that vaccine uptake increased in the analyzed period in the examined population after the introduction of mandatory pneumococcal vaccination. There was also a change in preference for the most commonly used formulation to PCV13 in favor of PCV10. However, there was no change in the frequency of discontinuation of a commenced vaccination schedule. Differences were also shown between vaccination rates in urban and rural outpatient clinics.

In Poland, in 2023, in the first year of life, vaccinations against tuberculosis, diphtheria, tetanus, pertussis (3-in-1), *Haemophilus influenzae* type B, poliomyelitis, hepatitis B, and pneumococcal (from 2017) and rotavirus infections (from 2021) are mandatory (free of charge). Before the introduction of mandatory pneumococcal vaccination, state institutions did not record data on vaccination rates, so it is not possible to compare the percentage of unvaccinated children from the survey (27.8%) to nationwide data. After the introduction of compulsory vaccination, registration began, and the percentage of unvaccinated children in the country, in 2017–2018, was 3.8% [7], which is consistent with the results of this survey (3.3%). This shows a significant increase in pneumococcal vaccine uptake among Polish children. An analogous trend has been observed, for example, in Bulgaria. PCV10 was introduced to the vaccination calendar in that country in 2010, and the percentage of vaccinated children increased from 69% in 2010 to 94% in 2011 [8,15].

Acceptance of vaccinations is a complex phenomenon, and receiving any vaccination depends on many factors. Studies have shown evidence of the effectiveness of several patient-centered interventions. These include making vaccinations more accessible, reducing their cost, reminders to vaccinate, and requiring vaccinations for enrollment in schools or other institutions [16]. The introduction of mandatory pneumococcal vaccination has had several effects. First, the financial barrier was removed, as the vaccination has since become free for all children born since 2017 [17]. It has been proven many times in the literature that free vaccination translates into a higher number of vaccinated individuals, and the cost of the vaccine is one of the more common reasons used to justify not vaccinating [18,19,20]. Second, the introduction of mandatory vaccination has also undoubtedly increased its popularity. Information campaigns in the media have made some parents aware of this vaccination. Parents were more likely to discuss vaccination with their doctor or seek information on their own, and this could ultimately translate into a positive decision to vaccinate their children. Studies confirm that increasing awareness of a particular vaccination, as well as popularizing knowledge about it, significantly increases the number of vaccinated patients [21]. On the other hand, it is also important to keep in mind the negative consequences of introducing mandatory vaccination. Willingness to receive compulsory vaccination is closely linked to trust in the authorities who introduce such an obligation. This is related to the history of introducing vaccination and the attitude of the community towards limiting their freedom of choice in favor of their own health and that of the population [22]. Moreover, as a result of reactance, forcing a parent to vaccinate a child may result in a desire to do the opposite. When giving a child a mandatory vaccination, they will decline another vaccination that they would normally have their child receive. This is what has been observed in Germany, where vaccine uptake for other diseases declined after measles vaccination became mandatory in 2020. A survey confirmed that compulsory measles vaccination in some social groups has triggered resistance to pneumococcal vaccination and the 6-in-1 combination vaccine [23]. Similar observations were made in another study by Léna G. Dietrich et al. among Swiss healthcare workers, where they were mostly opposed to mandatory vaccination both in the general population against measles and among healthcare workers against influenza. Some employees declared that they would resign if the obligation was introduced at their workplace [24]. Nevertheless, mandatory vaccination, in general, increases the percentage of vaccinations administered and thus improves public health [21]. Some parents, however, decide not to vaccinate their children despite the introduced obligation. A study by Cooper et al. divided parents who refuse vaccination into two groups: “Neoliberal logic” and “Social exclusion.” The first includes well-educated people from higher-income countries who value their individuality and independence to the point of distrusting the “system,” and thus resist vaccinating their children. The second group includes poorer people who are more excluded from society on many levels and see universal vaccination as a social construct from which they want to isolate themselves [25]. Nevertheless, trust in vaccination has been shown to be positively correlated with trust in science in general, and negatively correlated with belief in horoscopes [26]. This confirms the fact that better awareness of science, and thus vaccination, positively influences the acceptance of vaccination. To reach those parents who, for various reasons, are not convinced about vaccination, it seems effective to raise awareness and dispel doubts about immunization [27]. In Poland, most people approve of mandatory vaccinations, and some also support imposing sanctions on parents who evade vaccinating their children [28,29]. If mandatory vaccination were not widely accepted, the increase in the percentage of vaccinated children in the survey conducted would not have been so noticeable. An important element affecting the implementation of mandatory vaccinations is the government’s policy, which varies from country to country. A parent’s failure to comply with the obligation can prevent their child from being admitted to kindergarten or school, deprive parents of financial or tax benefits, or expose them to financial sanctions or even imprisonment [30]. However, in addition to legal provisions, the inevitability of the penalties provided for depriving a child of vaccination is also important—according to the classification proposed by Attwell et al., Poland is close to a system of “informal nonenforcement,” in which theoretically there is an obligation but compliance with it in practice is rarely enforced [31]. The complicated course of legal action and the possibility of dragging out the procedure means that a possible fine is imposed late and only in about 10% of cases of vaccination evasion [32]. This, in turn, makes the obligation likely to be ignored by parents, particularly if they accept a priori that they will pay the fine as the price for standing by their views.

Failure to complete a schedule that has been started is also a significant problem. In this study, it was observed that 11.8% of children started but did not complete the pneumococcal vaccination schedule. This percentage did not differ significantly between before and after the introduction of free vaccinations, at 11.8% and 11.9%, respectively. After analyzing patients who received at least one dose of pneumococcal vaccination, it was observed that the percentage of discontinued schedules for PCV10 decreased from 16.6% to 11.7%. The difference is on the verge of statistical significance but it makes it possible to observe a trend and further observations are required to make more conclusions. Completion of the vaccination schedule is important because of the protective potential. The number of PCV doses needed to achieve satisfactory immunity varies depending on the child’s age at which vaccination was started. Since IPD is most threatening to the youngest children, vaccination should be started as early as possible. Failure to administer all required primary doses decreases and/or shortens immunity against IPD, as does skipping a booster dose [33,34]. Completion of the vaccination schedule is affected by a great many factors including the number of doses required. Previous studies have shown that the greater the number of doses of vaccine, the greater the chance of discontinuing vaccination. A study by Krishnarajah et al. and a similar study by Luna-Casas et al. found that, when vaccinated against rotavirus, children were less likely to receive all doses of a three-dose vaccine compared to a two-dose vaccine [35,36]. Similar observations were made for *N. meningitidis* type B vaccines, where greater flexibility in the use of the MenB-4C vaccine made it more likely to be administered in the full schedule compared to the MenB-Fhbp vaccine [37]. A similar relationship can be seen in this study, where PCV10, administered in a 2+1 schedule was interrupted slightly less often than PCV13 that had to be administered in principle in a 3+1 schedule, since the 2+1 schedule is a conditional alternative in its case. It should also be borne in mind that a reason for parents’ refusal to have their child receive subsequent doses of vaccination is a negative experience after previous doses. This includes adverse reactions such as fever, pain at the injection site, and also the child’s fear of needles [38]. Another reason for not completing the vaccination schedule also continues to be the intentional avoidance of subsequent doses resulting from the belief that the doses already received are sufficient, which is a misconception [39]. In addition, organizational problems may also play a role—some studies indicate that the distance from the outpatient clinic translating into transportation difficulties may also have an impact on discontinuing vaccinations [40]. On the other hand, it should be mentioned that sometimes the interruption of the schedule is unintentional and results from the patient forgetting the need for another dose of vaccine. In such cases, healthcare workers should notify the patient of the need to complete the vaccination. This task is facilitated by modern technology, in which the automation of this process makes it possible to effectively remind about a vaccination dose without burdening the medical staff [41]. Differences have also been observed between rural and urban areas. In rural outpatient clinics, 84.8% of children were vaccinated against *pneumococcus*, while in urban ones, the figure was 70.8%. Moreover, in urban areas, 13% of children started but did not complete the vaccination schedule, while in rural areas this number was 6.5%. Nationwide statistics do not provide information on the place of residence of unvaccinated children [7]. In contrast, in a survey of attitudes toward COVID-19 vaccination conducted in Poland, it was rural residents who were more likely to have concerns about the vaccine and show no willingness to receive it [42]. At the same time, other studies do not clearly show whether the location of the clinic in or outside an urban area affects vaccination [43]. What is emphasized, however, is the fact that the involvement of medical personnel in promoting vaccination, reminding people of their next appointments, and passing on reliable knowledge to parents has a great impact [16]. It is possible that, in rural clinics with fewer patients, doctors may spend more time with patients and thus encourage vaccination more effectively.

Before the introduction of mandatory vaccination, parents were more likely to opt for PCV13 compared to PCV10, at a rate of 72.3% vs. 27.7%, respectively. Since parents were paying the cost of the vaccine anyway, they would be opting for the one with a broader spectrum. After the introduction of compulsory vaccination, this proportion reversed and most children were now vaccinated with PCV10—80.1% vs. 19.9%. Pneumococcal vaccination has been made compulsory in many European countries and, in most cases, vaccination is provided with PCV13 in a 2+1 schedule [44]. The introduction of vaccination has changed the distribution of serotypes that cause IPD [44,45,46]. In countries where PCV10 was used, the incidence of IPD caused by serotypes 3, 6A, and 19A contained in PCV13 but not in PCV10 increased. The increase is particularly noticeable for serotypes 3 and 19A [44,47]. In Poland, the number of IPD cases caused by serotypes contained in PCV10 has decreased every year since 2017, and, as in other countries, they are being replaced by non-vaccine serotypes and serotypes 3, 6A, and 19A, found in PCV13 but not in PCV10. Especially in the group of children under 5 years of age, the percentage of IPDs caused by serotype 19A has been increasing in recent years. That percentage was 11.54%, 34.43%, and 32.95% in 2020, 2021, and 2022, respectively [48,49]. In the analyzed population, the vast majority of children have been vaccinated with PCV10 since 2017, which is consistent with the observed trend of increase in the percentage of serotypes not included in PCV10. Data from European countries show that the introduction of mandatory pneumococcal vaccination has reduced the incidence of IPD. This effect was demonstrated for both PCV10 and PCV13 [50,51]. The difference is seen in the case of serotypes 6C and 19A, where PCV10 offers no significant protection. This could explain why, in countries using mainly PCV10 instead of PCV13, the percentage of IPD caused by this serotype increases. Also, data from outside Europe seem to confirm the superiority of PCV13 in protecting against the serotypes responsible for most IPD cases. In Canada, PCV13 showed about twice as much protection against serotypes in each of the analyzed regions of the country [52]. Protection against serotype 19A may be a factor responsible for reducing the number of antibiotic-resistant pneumococcal strains [53]. On the other hand, in systematic reviews, such conclusions are drawn with greater caution due to the wide variation in data in individual regions [54]. PCV10 induces cross-immunity against serotype 19A but, unlike PCV13, it does not reduce the carriage of this bacterium, thus providing less effective protection against this serotype [55].

Data from Poland may also indicate a positive impact of mandatory vaccinations on the incidence of IPD among children. In the data presented by the National Institute of Hygiene in 2014–2016, the incidence of pneumococcal meningitis and pneumococcal encephalitis among children aged 0–4 years was 1.07, 1.05, and 1.28 per 100,000, respectively. [56,57,58] From 2017 to 2021, a decreasing trend in incidence was observed, which was 0.85, 1.26, 0.94, 0.26, and 0.75, respectively [59,60,61,62,63]. Nevertheless, the last two years of the report, i.e., 2020 and 2021, should be treated with caution due to the current COVID-19 pandemic and restrictions in force that could definitely affect the above values [64]. Unfortunately, the analyzed data do not include information on the most common serotypes causing IPD.

The authors are aware of the limitations of the present study, namely the selection of a sample group that is not representative of the Polish population, and therefore further observations on a larger group of patients are necessary. Also, the reason for discontinuing vaccination is not known.

In conclusion, the introduction of mandatory pneumococcal vaccination has contributed to a significant increase in the percentage of vaccinated children. It also contributed to a change in the trend of the formulation used with a predominance toward PCV10. Better vaccination coverage will most likely translate into reduced mortality among infants and children up to 5 years of age. This is one of the assumptions of goal 3.3 of the Sustainable Development Goals created by the United Nations. Making vaccines free of charge is also part of the implementation of goal 3.8, which concerns ensuring financially accessible modern medical methods [65].

## 5. Conclusions

The introduction of free mandatory pneumococcal vaccination has increased the percentage of vaccinated children but has not affected the percentage of uncompleted vaccination schedules. Introducing free-of-charge compulsory pneumococcal vaccination with the PCV10 vaccine has resulted in changes in the contribution of specific pneumococcal serotypes to the etiology of invasive pneumococcal disease in Poland.

## Figures and Tables

**Table 1:** The “vaccinated completely” group criteria for children born in 2015 and 2016.

Patient’s Age	PCV10 ^1^	PCV13 ^2^
From 6 weeks to 6 months of age	3+1 1. Three primary doses with an interval of at least 1 month between doses. 2. A booster dose at least after the last primary dose and from the age of 9 months.	3+1 1. Three primary doses with an interval of at least 1 month between doses. 2. A booster dose is recommended between 11 and 15 months of age.
7 months–11 months	2+1 1. Two primary doses with an interval of at least 1 month between doses. 2. A booster dose in the second year of life, at least 2 months after the last primary dose.	2+1 1. Two primary doses with an interval of at least 1 month between doses. 2. A booster dose is recommended in the second year of life
12 months–23 months	2+0 1. Two doses with an interval of at least 2 months between doses.	2+0 1. Two doses with an interval of at least 2 months between doses
24 months–5 years		1+0 1. One single dose
5 years and above	Not registered for use.	

^1^ PCV10—10-valent conjugate pneumococcal vaccine; ^2^ PCV13—13-valent conjugate pneumococcal vaccine.

**Table 2:** The “vaccinated completely” group criteria for children born in 2017 and 2018.

Patient’s Age	PCV10 ^1^	PCV13 ^2^
From 6 weeks to 6 months of age	3+1 1. Three primary doses with an interval of at least 1 month between doses. 2. A booster dose at least 6 months after the last primary dose and from the age of 9 months. OR 2+1 1. Two primary doses with an interval of at least 2 months between doses. 2. A booster dose at least 6 months after the last primary dose and form the age of 9 months.	3+1 1. Three primary doses with an interval of at least 1 month between doses. 2. A booster dose is recommended between 11 and 15 months of age. OR 2+1 1. Two primary doses with an interval of at least 2 months between doses. 2. A booster dose is recommended between 11 and 15 months of age.
7 months–11 months	2+1 1. Two primary doses with an interval of at least 1 month between doses. 2. A booster dose in the second year of life, at least 2 months after the last primary dose.	2+1 1. Two primary doses with an interval of at least 1 month between doses. 2. A booster dose is recommended in the second year of life
12 months–23 months	2+0 1. Two doses with an interval of at least 2 months between doses.	2+0 1. Two doses with an interval of at least 2 months between doses
24 months–5 years		1+0 1. One single dose
5 years and above	Not registered for use.	

^1^ PCV10—10-valent conjugate pneumococcal vaccine; ^2^ PCV13—13-valent conjugate pneumococcal vaccine.

**Table 3:** Characteristics of the sample group.

Patient		Total Population *N* (%)	2015 and 2016 Age Group N (%)	2017 and 2018 Age Group *N* (%)	*p*
Gender	Male	763 (47.8)	387 (46.5)	445 (53.5)	0.525
	Female	832 (52.2)	368 (48.2)	395 (51.8)	
Place	Urban area	1318 (82.6)	630 (47.8)	688 (52.2)	0.417
	Rural area	277 (17.4)	125 (45.1)	152 (54.9)	

**Table 4:** Vaccination status and vaccine used.

Patient		Total Population *N* (%)	2015 and 2016 Age Group *N* (%)	2017 and 2018 Age Group *N* (%)	*p*	Cramér’s V
Vaccinated against *pneumococcus*	Yes	1168 (73.2)	456 (60.4)	712 (84.8)	<0.001	0.275
	No	427 (26.8)	299 (39.6)	128 (15.2)		
Vaccination schedule	Completed	1168 (73.2)	456 (60.4)	712 (84.8)	<0.001	0.346
	Not completed	189 (11.8)	89 (11.8)	100 (11.9)		
	No vaccination	238 (15.0)	210 (27.8)	28 (3.3)		
Product (*N* = 1357)	PCV10 ^1^	801 (59.0)	151 (27.7)	650 (80.1)	<0.001	0.521
	PCV13 ^2^	556 (41.0)	394 (72.3)	162 (19.9)		

^1^ PCV10—10-valent conjugate pneumococcal vaccine; ^2^ PCV13—13-valent conjugate pneumococcal vaccine.

**Table 5:** Pneumococcal vaccination by gender, place, and vaccine type.

Variable		Pneumococcal Vaccination *N* (%)			Completed Schedule *N* (%)			
		Yes	No	*p*	Vaccinated Completely	Not Fully Vaccinated	Not Vaccinated	*p*
Gender	Male	608 (73.1)	224 (26.9)	0.886	608 (73.1)	99 (11.9)	125 (15.0)	0.989
	Female	560 (73.4)	203 (26.6)		560 (73.4)	90 (11.8)	113 (14.8)	
Place	Urban area	933 (70.8)	385 (29.2)	<0.001	933 (70.8)	171 (13.0)	214 (16.2)	<0.001
	Rural area	235 (84.8)	42 (15.2)		235 (84.8)	18 (6.5)	24 (8.7)	
Vaccine	PCV10 ^1^	—	—	—	700 (87.4)	101 (12.6)	—	0.093
	PCV13 ^2^	—	—		468 (84.2)	88 (15.8)	—	

^1^ PCV10—10-valent conjugate pneumococcal vaccine; ^2^ PCV13—13-valent conjugate pneumococcal vaccine.

**Table 6:** Pneumococcal vaccination in 2015–2016 and 2017–2018 by gender, locality, and vaccine.

Variable			Pneumococcal Vaccination *N* (%)			Completed Schedule *N* (%)			
			Yes	No	*p*	Vaccinated Completely	Not Fully Vaccinated	Not Vaccinated	*p*
Gender	2015–2016	Male	228 (58.9)	159 (41.1)	0.393	228 (58.9)	49 (12.7)	110 (28.4)	0.635
		Female	228 (62.0)	140 (38.0)		228 (62.0)	40 (10.9)	100 (27.2)	
	2017–2018	Male	380 (85.4)	65 (14.6)	0.532	380 (85.4)	50 (11.2)	15 (3.4)	0.817
		Female	332 (84.1)	63 (15.9)		332 (84.1)	50 (12.7)	13 (3.3)	
Place	2015–2016	Urban area	364 (57.8)	266 (42.2)	<0.001	364 (57.8)	77 (12.2)	189 (30.0)	0.003
		Rural area	92 (73.6)	33 (26.4)		92 (73.6)	12 (9.6)	21 (16.8)	
	2017–2018	Urban area	569 (82.7)	119 (17.3)	<0.001	569 (82.7)	94 (13.7)	25 (3.6)	0.002
		Rural area	143 (94.1)	9 (5.9)		143 (94.1)	6 (3.9)	3 (2.0)	
Vaccine	2015–2016	PCV10 ^1^	—	—	—	126 (83.4)	25 (16.6)	—	0.930
		PCV13 ^2^	—	—		330 (83.8)	64 (16.2)	—	
	2017–2018	PCV10 ^1^	—	—	—	574 (88.3)	76 (11.7)	—	0.279
		PCV13 ^2^	—	—		138 (85.2)	24 (14.8)	—	

^1^ PCV10—10-valent conjugate pneumococcal vaccine; ^2^ PCV13—13-valent conjugate pneumococcal vaccine.

**Table 7:** Percentage of completed and uncompleted vaccination schedules in 2015–2016 vs. 2017–2018 depending on the vaccine used.

Vaccine	2015 and 2016 Age Group *N* (%)		2017 and 2018 Age Group *N* (%)		*p*
	Schedule Completed	Schedule Not Completed	Schedule Completed	Schedule Not Completed	
PCV10 ^1^	126 (83.4)	25 (16.6)	574 (88.3)	76 (11.7)	0 = 0.105
PCV13 ^2^	330 (83.8)	64 (16.2)	138 (85.2)	24 (14.8)	0 = 0.675

^1^ PCV10—10-valent conjugate pneumococcal vaccine; ^2^ PCV13—13-valent conjugate pneumococcal vaccine.

## Data Availability

The heads of the primary care facilities where the data were collected agreed only to the collective presentation of the analysis results without the possibility of publishing the full database.
